# Development of a predictive framework for ovarian reserve decline based on pelvic microbiota dysbiosis

**DOI:** 10.1007/s13167-025-00417-4

**Published:** 2025-08-09

**Authors:** Jie Luo, Lili Cao, Junnan Li, Tao Zhang, Ketan Chu, Wenxian Xu, Zaigui Wu, Wanting Shen, Jianhong Zhou, Chanyuan Li

**Affiliations:** 1 Women’s Hospital, Zhejiang University School of Medicine, Hangzhou, 310006 Zhejiang China; 2 Hangzhou Linping District Maternal & Child Health Care Hospital, Hangzhou, Zhejiang 311199 China; 3 Department of Obstetrics and Gynecology, The First People’s Hospital of Yongkang, Yongkang, Zhejiang 321000 China; 4 Division of Engineering in Medicine, Brigham and Women’s Hospital, Harvard University, Boston, 02139 MA USA

**Keywords:** Predictive preventive personalized medicine (PPPM / 3PM), Pelvic microbiota dysbiosis, Diminished ovarian reserve, Microbial biomarker, Risk stratification, Microbiota profiling, Personalized reproductive diagnostics, Reproductive phenotyping, Suboptimal reproductive health, Integrated prediction model

## Abstract

**Background:**

Diminished ovarian reserve (DOR) is increasingly recognized as a multifactorial condition, not solely related to aging. Emerging evidence suggests that environmental and biological factors, including the pelvic microbiota, may influence ovarian function across different age groups. In this study, we examined the association between pelvic microbiota dysbiosis and DOR, with the broader goal of identifying early microbiota-based markers to support predictive diagnosis, preventive strategies, and personalized reproductive care.

**Methods:**

Ascitic fluid samples were collected from women with normal ovarian reserve and those diagnosed with DOR. Microbial profiling was performed using 16S ribosomal RNA (rRNA) gene sequencing to compare the composition and diversity of the pelvic microbiota between the two groups. A multivariable predictive model was constructed by combining key microbial genera with clinical indicators such as body mass index (BMI), aiming to support early risk estimation of DOR.

**Results:**

Microbial analysis revealed a significantly higher abundance of *Capnocytophaga* in the DOR group compared to controls, suggesting its potential role as a microbial marker of diminished ovarian reserve. The predictive model integrating microbial and clinical data demonstrated moderate accuracy, with an area under the curve (AUC) of 0.88 ± 0.16.

**Conclusions:**

Women with a BMI ≥ 24.0 face an increased risk of ovarian function decline. If pelvic microbiota profiling further reveals dysbiosis, particularly *Capnocytophaga* enrichment, early microbial screening and individualized probiotic treatment with *Lactobacillus* or *Bifidobacterium* may be warranted. This strategy embodies the core principles of predictive, preventive, and personalized medicine (PPPM/3PM).

## Introduction

Diminished ovarian reserve (DOR) [1–4] is a prevalent condition that significantly impacts reproductive health, leading to compromised fertility, hormonal imbalance, and a variety of related health concerns [5–8]. As the age of women seeking fertility treatments continues to rise, the incidence of ovarian dysfunction has become a growing clinical challenge. Despite significant advances in diagnostic techniques and treatment options, the underlying pathophysiology remains incompletely understood, and current approaches are largely reactive, focusing on symptomatic management rather than early detection or prevention. While much of the focus has been placed on genetic and endocrine factors, emerging studies suggest that microbiota, particularly those residing in the pelvic region, may influence ovarian physiology and reproductive outcomes [9–12].

Several factors have been implicated in diminished ovarian reserve, including genetic predisposition, autoimmune conditions, lifestyle factors, and environmental exposures. However, one of the most compelling emerging factors is the influence of infections and inflammation on ovarian reserve. Infections of the reproductive system, particularly chronic pelvic inflammatory disease (PID), have been implicated in diminished ovarian reserve through mechanisms such as chronic inflammation, immune system activation, and hormonal disruption [13–15]. Increasingly [16], subclinical microbial dysbiosis, rather than overt infection, is being considered a potential contributor to ovarian dysfunction. Low-grade inflammation driven by pelvic microbial imbalance may represent an overlooked risk factor for suboptimal reproductive health. This underscores the need for predictive assessments and stratified prevention strategies based on individual microbial profiles, a direction increasingly emphasized in personalized and preventive reproductive medicine [17–19].

The relationship between pelvic microbiota and ovarian reserve remains an area of active investigation. While research has predominantly focused on the vaginal and gut microbiota, their relevance to ovarian function is still being clarified [20–22]; little is known about the role of ascitic microbiota in influencing ovarian health. Ascitic fluid, which can accumulate in the pelvic cavity due to various reproductive disorders, provides a unique opportunity to examine the peritoneal microenvironment and its potential influence on ovarian health. As a relatively underexplored niche, the ascitic microbiome may reflect deeper dysregulation within the pelvic ecosystem. Recent studies [23–25] have suggested that microbial dysbiosis may lead to systemic inflammation, oxidative stress, and disruption of hormonal pathways, all of which could contribute to the onset of diminished ovarian reserve. These findings support further investigation of pelvic microbial profiles in ascitic fluid as potential early indicators of ovarian dysfunction and as candidate targets for stratified prevention in reproductive medicine.

This study aims to bridge this gap by investigating the correlation between ascitic microbiota composition and ovarian reserve status. We will analyze ascitic fluid microbiota from 45 patients with varying degrees of ovarian reserve, performing microbial 16S ribosomal RNA (rRNA) gene sequencing to identify microbial profiles and correlating these findings with ovarian reserve markers and inflammatory indicators. Our goal is to uncover microbial signatures with predictive value for early-stage DOR and explore their potential utility in risk stratification and personalized reproductive health management. By elucidating the role of the pelvic microbiome in ovarian reserve, this study may inform the development of microbiota-based diagnostic approaches and lay the groundwork for future preventive strategies.

## Methods

### Study design

This study is a retrospective observational study designed to investigate the correlation between ascitic microbiota and ovarian reserve. We aim to analyze the microbial community composition in ascitic fluid samples from patients with different ovarian reserve. Additionally, we will explore the relationship between pelvic microbiota and ovarian reserve, based on retrospective clinical data, and assess its potential as a predictive marker for reproductive health.

### Sample collection

A total of 45 female patients will be randomly selected for the study, and ascitic fluid will be collected during gynecological laparoscopic surgery. Ascitic fluid (10 ml) will be collected in centrifuge tubes under sterile conditions, stored at − 80 °C, and used for subsequent sequencing. Importantly, all included ascitic fluid samples were collected from patients who did not present with malignant ascites or systemic inflammatory conditions. The presence of trace or small-volume pelvic ascitic fluid is commonly observed during gynecologic laparoscopy for benign, non-inflammatory conditions and is considered physiologically or locally reactive in origin. Patients with any diagnosis of malignancy, liver cirrhosis, pelvic inflammatory disease, or systemic infections were excluded to minimize confounding factors associated with pathological ascites. Informed consent was obtained from all participants, and the study protocol was approved by the institutional review board (IRB-20240099-R). Patient information, including age, complete blood count, C-Reactive Protein (CRP) levels, hormonal profiles (follicle-stimulating hormone (FSH), luteinizing hormone (LH), estradiol (E2), anti-müllerian hormone (AMH), vaginal smear, and obstetric history (including number of pregnancies, deliveries, and miscarriages) will be recorded. Patients will be divided into two age groups (≤ 40 years and > 40 years) for 16S rRNA analysis. Subsequently, patients were categorized into the DOR group and control group based on AMH with a cutoff value of 1.1 ng/ml for clinical analysis. The control group included 23 patients with AMH ≥ 1.1 ng/ml, while the DOR group consisted of 22 patients with AMH < 1.1 ng/ml [3].

#### *Microbial sequencing *[26, 27]

Ascitic fluid samples were snap frozen and stored at − 80 °C after collection. Bacterial DNA was isolated from the samples using a DNeasy PowerSoil kit (Qiagen, Hilden, Germany) following the manufacturer’s instructions. DNA concentration and integrity were measured by a NanoDrop 2000 spectrophotometer (Thermo Fisher Scientific, Waltham, MA, USA) and agarose gel electrophoresis, respectively. PCR amplification of the V3-V4 hypervariable regions of the bacterial 16S rRNA gene was carried out in a 25 μl reaction using universal primer pairs (343F: 5′-TACGGRAGGCAGCAG-3′; 798R: 5′-AGGGTATCTAATCCT-3′). The reverse primer contained a sample barcode, and both primers were ligated to an Illumina sequencing adapter.

The amplicon quality was visualized using gel electrophoresis. The polymerase chain reaction (PCR) products were purified with Agencourt AMPure XP beads (Beckman Coulter Co., USA) and quantified using Qubit dsDNA assay kit. The concentrations were then adjusted for sequencing. Sequencing was performed on an Illumina NovaSeq6000 with two paired-end read cycles of 250 bases each. (Illumina Inc., San Diego, CA; OE Biotech Company; Shanghai, China).

Raw sequencing data were in FASTQ format. Paired-end reads were then preprocessed using Cutadapt software to detect and cut off the adapter. After trimming, paired-end reads were filtered for low-quality sequences, denoised, merged, and detected, and cut off the chimera reads using DADA2 with the default parameters of QIIME2 (2020.11). At last, the software output the representative reads and the amplicon sequence variant (ASV) abundance table. The representative read of each ASV was selected using the QIIME 2 package. All representative reads were annotated and blasted against Silva database Version 138 (16 s/18 s/ITS rDNA) using q2-feature-classifier with the default parameters. The microbial diversity in ascitic fluid samples was estimated using the alpha diversity that includes Simpson index. The Bray–Curtis distance matrix performed by QIIME software was used for Bray–Curtis principal coordinates analysis (PCoA) and phylogenetic tree construction. The 16S rRNA gene amplicon sequencing and analysis were conducted by OE Biotech Co., Ltd. (Shanghai, China).

#### Statistical analysis

Statistical analysis was performed using SPSS 23.0 software and Python. To compare the basic characteristics between groups, if the data conformed to a normal distribution and the variance was uniform, the results were expressed as the mean ± standard deviation (SD), and the *t*-test was used. Otherwise, a nonparametric test was applied, and the results were expressed as the median (P25–P75) values. The chi-square test (*χ*^2^ test) was used for categorical variables, and results were expressed as the number of cases and percentages. Given that the bacterial abundance data were not normally distributed, their concentrations were described as median and interquartile ranges (P25–P75), and the Mann–Whitney *U* test was used to compare differences between groups. Logistic regression analysis was performed to assess the association between bacterial abundance and DOR, with each genus categorized into low and high abundance based on the median value across all subjects. Crude odds ratios (ORs) with 95% confidence intervals (95% CIs) were calculated. Model performance was evaluated using tenfold cross-validation. Receiver operating characteristic (ROC) curves were generated, and the area under the curve (AUC) was calculated using the ROC curve and AUC functions from the sklearn metrics module in Python. The accuracy score function was also used to assess overall model accuracy.

## Results

### Microbial composition in ascitic fluid

**Fig. 1:**
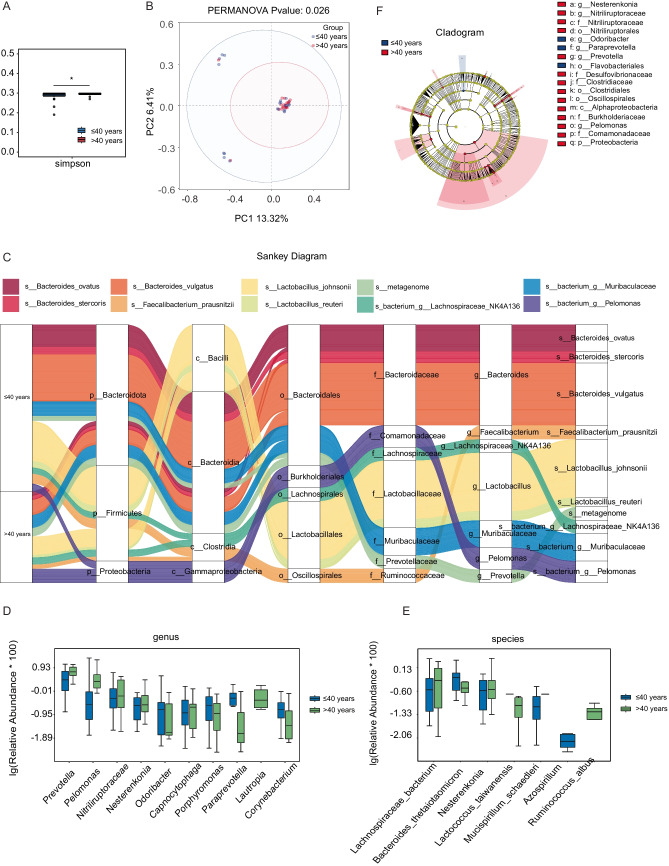
Microbial composition and diversity of ascitic fluid stratified by age. **A** Alpha diversity analysis showed differences in microbial diversity between two groups, using the Simpson index (*P* = 0.0344 < 0.05, *). **B** PCoA revealed distinct clustering of microbial communities between the groups, based on Bray–Curtis distance (*Permanova P* = 0.026). **C** Sankey diagram illustrating the taxonomic distribution of microbiota from phylum to species level. **D** Differentially expressed microbial genera (top 10) were compared between the two groups using the Wilcoxon *t* test (*P* < 0.05). **E** Differentially expressed microbial species (top 10) were compared between the two groups using the Wilcoxon *t* test (*P* < 0.05). **F** LEfSe analysis identified microbial taxa significantly distinguishing the groups

To investigate the potential influence of pelvic microbiota on ovarian reserve, ascitic fluid samples were collected from 45 patients undergoing surgery for benign gynecological tumors. Microbial composition and diversity were analyzed using 16S rRNA sequencing. Given the established association between age and ovarian reserve, patients were initially stratified into two age groups (≤ 40 years and > 40 years) to explore potential age-related shifts in microbiota that may contribute to diminished ovarian reserve. To assess overall microbial diversity, we conducted alpha diversity analysis, which measures species richness and evenness within each sample. The results showed that the Simpson index was significantly lower in the younger age group (age ≤ 40 years) *P* = 0.0344, indicating reduced microbial diversity (Fig. 1A). PCoA was then applied to visualize beta diversity, which evaluates differences in microbial community structure between samples. The PCoA plot, based on the Bray–Curtis distance, revealed distinct clustering between the two age groups, indicating significant microbial compositional differences (*Permanova P* = 0.026) (Fig. 1B). To further characterize the microbial composition of ascitic fluid, we constructed a Sankey diagram to visualize taxonomic distribution at different classification levels, from phylum to species (Fig. 1C). The dominant phyla across all samples included *Bacteroidota*,* Firmicutes*, and *Proteobacteria*. At the genus level, *Bacteroides*, *Faecalibacterium*, *Lactobacillus*, *Muribaculaceae*, *Prevotella*, and *Pelomonas* were predominant, with notable differences observed between the two groups. We further analyzed the differentially expressed microbial genera and species between the two groups (Fig. 1D, E). At the species level (Fig. 1E), *Lachnospiraceae bacterium*, *Nesterenkonia*, *Lactococcus taiwanensis*, and *Ruminococcus albus* were significantly overrepresented in the older age group. These findings suggest that specific microbial species may be associated with ovarian function alterations related to aging. To identify taxa with significant differential abundance between the groups, we performed linear discriminant analysis effect size (LEfSe) analysis (Fig. 1F). This method uses linear discriminant analysis (LDA) to determine the effect size of each taxon, allowing us to identify taxa that most strongly distinguish between the groups. The LEfSe results revealed several microbial genera and species that were significantly different between the younger and older age groups. Specifically, taxa such as *Proteobacteria*, *Pelomonas*, and *Prevotella* were significantly enriched in the older age group, with an LDA score greater than 10^4^. These findings suggest potential microbial signatures that may be linked to ovarian function.

This preliminary stratification highlights that age-related changes in the ascitic microbiome may reflect early shifts in ovarian function. However, chronological age alone may not fully capture the complexity of reproductive decline. Building on this observation, we propose that specific microbial signatures could serve as more sensitive and earlier indicators of DOR than conventional age or hormonal markers. Integrating these microbial profiles with clinical data holds potential for identifying high-risk individuals before symptoms appear, enabling timely lifestyle or probiotic interventions and promoting a shift toward proactive, personalized reproductive care.

### Clinical and microbiota profiles in the study cohort

In the first part, we analyzed the ascitic microbiota based on age groups to assess general microbial differences across reproductive stages. To further clarify the association between pelvic microbiota and ovarian reserve, we then stratified patients by AMH levels. This approach facilitated the identification of DOR-related microbial features and enabled further clinical analysis of microbiome–ovarian function associations. The clinical characteristics of the study cohort are summarized in Table 1; a total of 45 participants were included, with 23 in the control group (AMH ≥ 1.1 ng/ml) and 22 in the DOR group (AMH < 1.1 ng/ml). Age was significantly higher in the DOR group (44.91 ± 6.63 years) compared to the control group (31.39 ± 5.92 years), with a *P* value of 0.000, indicating a strong association between age and ovarian reserve. The levels of FSH were also significantly elevated in the DOR group [12.95 (6.14–33.25) IU/l] compared to the control group [5.13 (3.74–6.63)] IU/l, with a *P* value of 0.000, suggesting a disrupted hormonal balance in the DOR group. Moreover, the gravity and parity of participants were significantly higher in the DOR group compared to the control group, with *P* values of 0.000. This increase may be attributed to the older age of participants in the DOR group, which is associated with a higher number of pregnancies and births. In terms of BMI (body mass index), the DOR group exhibited a significantly higher value [23.34 (21.42–24.71)] kg/m^2^ compared to the control group [20.58 (19.78–24.38)] kg/m^2^, with a *P* value of 0.038. This suggests that body weight may have an impact on ovarian function, with higher BMI potentially contributing to reduced ovarian reserve in the DOR group. Though no significant differences were observed between the two groups for several clinical parameters, including white blood cell (WBC), red blood cell (RBC), platelet (PLT), and CRP, certain immune-related markers, such as eosinophils (EOS) and basophils (BASO), exhibited trends toward differences that did not reach statistical significance. Furthermore, the prevalence of abnormal vaginal smear tests was higher in the DOR group (*P* = 0.057), suggesting a potential trend toward increased vaginal microbiota dysbiosis in individuals with diminished ovarian reserve.

**Table 1:** Characteristics of the study cohort. Data are presented as median (P25–P75) or mean ± SD, depending on data distribution. DOR group: AMH < 1.1 ng/ml (*n* = 22); control group: AMH ≥ 1.1 ng/ml (*n* = 23) (symbols represent significance levels: *P < 0.05; **P < 0.01; **P < 0.001)

	DOR	Control	*P* value
Age, year	44.91 ± 6.63	31.39 ± 5.92	0.000**
WBC	4.70 (3.78–6.53)	5.60 (4.80–7.40)	0.059
RBC	4.32 ± 0.49	4.31 ± 0.43	0.909
PLT	239.00 (199.00–296.75)	252.00 (226.00–348.00)	0.204
PLT-LCC	10.10 (9.45–10.55)	10.20 (9.00–10.90)	0.964
NEU	60.06 ± 8.35	60.81 ± 9.08	0.774
EOS	1.00 (0.48–2.68)	2.00 (0.80–3.40)	0.128
BASO	0.48 ± 0.30	0.64 ± 0.36	0.128
LYM	30.79 ± 7.26	29.37 ± 8.20	0.542
MON	6.79 ± 2.69	6.77 ± 1.37	0.979
CRP	0.70 (0.30–1.48)	1.10 (0.40–1.80)	0.446
LH	8.34 (5.54–33.25)	9.29 (4.61–14.60)	0.394
FSH	12.95 (6.14–33.25)	5.13 (3.74–6.63)	0.000**
E2	338.00 (157.75–449.25)	365.00 (187.00–687.00)	0.394
P	0.84 (0.59–2.01)	1.21 (0.83–5.89)	0.146
Gravity	3 (2–4)	0 (0–2)	0.000**
Parity	2 (1–2)	0 (0–1)	0.000**
BMI	23.34 (21.42–24.71)	20.58 (19.78–24.38)	0.038*
Vaginal smear test			0.057
Yes	10	4	
No	12	19	

These findings suggest that clinical and hormonal markers, such as age, FSH, gravity, parity, and BMI, are strongly associated with ovarian function. Age-related ovarian decline is well-documented, with higher FSH indicating reduced reserve. Additionally, BMI may influence hormonal balance and metabolic status, indirectly impacting ovarian health. The observed associations between these clinical markers and pelvic microbiota patterns related to aging may offer predictive insights into the onset of diminished ovarian reserve. Integrating microbiota analysis with conventional clinical indicators may enhance early risk stratification of DOR. This combined approach supports targeted prevention strategies, such as lifestyle modification or probiotic interventions in high-risk individuals, thereby contributing to personalized and anticipatory reproductive care.

Following the comparison of baseline clinical characteristics between the DOR and control groups, we listed the differentially abundant bacterial genera in Table 2. Notable shifts were observed in both pathogenic and commensal taxa between the groups. Among the pathogenic genera, *Capnocytophaga* showed a higher median abundance in the DOR group [4.00 (IQR) 3.40–4.00)] compared to the control group [3.23 (IQR 2.63–4.00)]. In contrast, the genera *Lautropia* [4.00 (IQR 4.00–4.00)], *Pelomonas* [2.51 (IQR 1.94–3.07)], and *Nesterenkonia* [4.00 (IQR 3.47–4.00)] had higher median abundances in the control group compared to the DOR group [4.00 (IQR 2.68–4.00), 1.63 (IQR 1.31–2.01), and 2.62 (IQR 2.38–4.00)]. These findings suggest that *Capnocytophaga* may be associated with diminished ovarian reserve, while *Lautropia*, *Pelomonas*, and *Nesterenkonia* are more abundant in individuals with normal ovarian reserve, which may indicate that these potentially pathogenic genera are less likely to be involved in ovarian dysfunction.

**Table 2:** The differential distribution of genera in the DOR and control group. Bacterial abundance is shown as − log_10_ (*X* + 0.0001); values are presented as mean ± SD and median (IQR), depending on data distribution (symbols represent significance levels: *P < 0.05; **P < 0.01; **P < 0.001)

	DOR		Control		*P* value
	Mean ± SD	Median (IQR)	Mean ± SD	Median (IQR)	
Pathogenic					
Capnocytophaga	3.66 ± 0.58	4.00 (3.40–4.00)	3.13 ± 0.80	3.23(2.63–4.00)	0.015*
Lautropia	3.51 ± 0.83	4.00 (2.68–4.00)	4.00 ± 0.00	4.00 (4.00–4.00)	0.004**
Pelomonas	1.79 ± 0.79	1.63 (1.31–2.01)	2.48 ± 0.71	2.51 (1.94–3.07)	0.006**
Nesterenkonia	2.93 ± 0.81	2.62 (2.38–4.00)	3.67 ± 0.62	4.00 (3.47–4.00)	0.001**
Commensal					
UCG-002	2.80 ± 0.96	2.76 (1.80–4.00)	3.35 ± 0.75	3.79 (2.58–4.00)	0.032*
UCG-005	3.78 ± 0.48	4.00 (3.88–4.00)	3.46 ± 0.67	3.73 (3.13–4.00)	0.031*
NK4A214_group	3.69 ± 0.60	4.00 (3.69–4.00)	3.40 ± 0.65	3.37 (3.12–4.00)	0.041*
0319-7L14	3.97 ± 0.12	4.00 (4.00–4.00)	3.76 ± 0.42	4.00 (3.51–4.00)	0.025*
Telmatospirillum	4.00 ± 0.00	4.00 (4.00–4.00)	3.92 ± 0.21	4.00 (4.00–4.00)	0.043*
Candidatus_Solibacter	3.95 ± 0.25	4.00 (4.00–4.00)	3.49 ± 0.66	4.00 (2.88–4.00)	0.003**
Nitriliruptoraceae	2.59 ± 0.95	2.46 (1.63–3.60)	3.22 ± 0.98	4.00 (2.20–4.00)	0.045*

Compared to the DOR group, the control group exhibited higher median abundances of *UCG-002* [3.79 (IQR 2.58–4.00)] and *Nitriliruptoraceae* [4.00 (IQR 2.20–4.00)]. Conversely, the DOR group showed elevated levels of several other commensal genera, including *UCG-005* [4.00 (IQR 3.88–4.00)], *NK4A214_group* [4.00 (IQR 3.69–4.00)], *0319-7L14* [4.00 (IQR 4.00–4.00)], *Telmatospirillum* [4.00 (IQR 4.00–4.00)], and *Candidatus_Solibacter* [4.00 (IQR 4.00–4.00)], all of which had lower median values in the control group. This shift in microbial composition suggests that the DOR-associated environment may favor the expansion of certain commensal taxa. While these bacteria are generally not considered pathogenic, their enrichment might represent a microbial response to underlying inflammation, hormonal dysregulation, or metabolic stress. Whether these changes serve a protective function or reflect early dysbiosis remains to be elucidated.

Interestingly, our findings suggest that diminished ovarian reserve is not necessarily associated with an increase in pathogenic bacteria. Instead, several commensal genera were more abundant in the DOR group. This unexpected shift in microbial composition may represent a compensatory or adaptive response to changes in the ovarian or pelvic microenvironment, rather than a consequence of overt microbial invasion. These results underscore the importance of further clinical and mechanistic investigations to clarify the functional role of commensal microbiota in ovarian health and their potential relevance in predictive modeling.

#### Association between altered microbiota and DOR risk

**Fig. 2:**
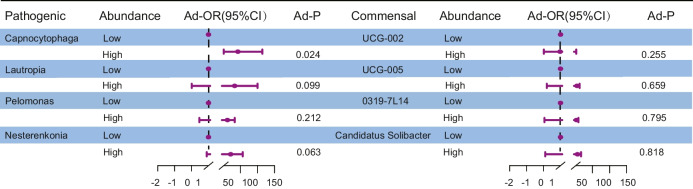
Multivariate logistic regression analysis showing the association between the relative abundance of bacterial genera and the likelihood of DOR. ORs were adjusted for age, BMI, and other covariates. The vertical dashed line indicates OR = 1.0

The relationship between the relative abundance of individual bacterial genera and the likelihood of DOR was assessed using multivariate logistic regression analysis, adjusting for relevant covariates. The results are visualized in Fig. 2. Among the taxa analyzed, high abundance of *Capnocytophaga* was significantly associated with an increased risk of DOR. Compared to individuals with low levels of *Capnocytophaga*, those with high levels had a markedly elevated likelihood of DOR (adjusted OR = 12.644; 95% CI, 1.399–114.279; *P* = 0.024). Although elevated levels of other potentially pathogenic bacteria, such as *Lautropia*, *Pelomonas*, and *Nesterenkonia*, also showed trends toward increased odds of DOR, these associations did not reach statistical significance (all *P* > 0.05). Similarly, the abundance of commensal taxa (*UCG-002*, *UCG-005*, *0319-7L14*, *Candidatus Solibacter*) was not significantly correlated with DOR risk. *Capnocytophaga* was identified as a potential microbial biomarker associated with diminished ovarian reserve, warranting further investigation to clarify its mechanistic role and predictive relevance.

#### Microbiota-based predictive modeling for ovarian reserve

**Fig. 3:**
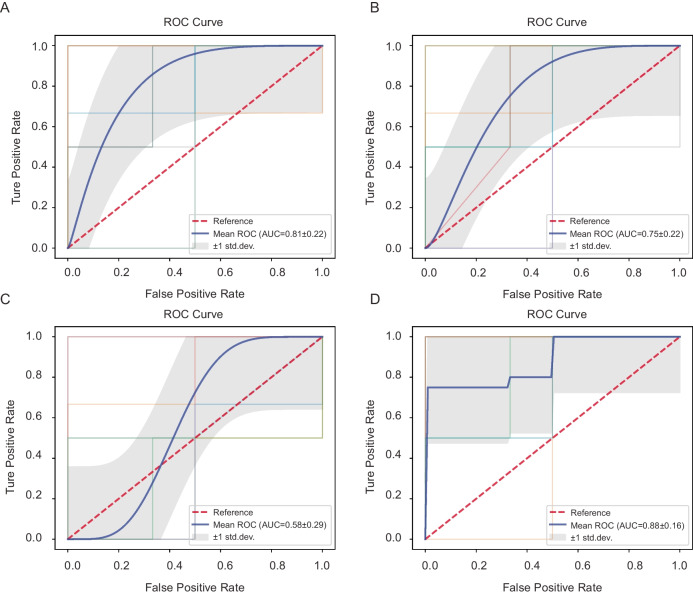
Predictive models for ovarian reserve risk based on microbial and clinical parameters: **A** model based on key microbial genera, **B** model based on BMI, **C** model based on inflammatory markers, **D** combined model of microbiota + BMI

To explore the potential of using pelvic microbiota and clinical parameters to predict the risk of DOR, we constructed multiple random forest classification models based on different feature sets, including eight key microbial genera, BMI, systemic inflammatory markers, and a combined model integrating microbial and metabolic features. The microbial genera were selected via multivariate logistic regression analysis (Fig. 2) based on their predictive contributions and biological plausibility. All models were trained using tenfold cross-validation to ensure robustness. Performance was assessed using ROC, with the AUC used as a measure of discriminative accuracy (Fig. 3).

The model based on key microbial genera showed moderate predictive power (AUC = 0.81 ± 0.22) (Fig. 3A), suggesting that certain taxa may be linked to the risk of DOR. This result indicates that microbial dysbiosis in the pelvic environment may serve as an early and specific signature for ovarian reserve estimation. In contrast, the BMI-only model exhibited moderate discriminative performance (AUC = 0.75 ± 0.22) (Fig. 3B), suggesting that metabolic status alone, as reflected by BMI, may be partially informative in predicting ovarian reserve. This finding is consistent with existing evidence that metabolic imbalance can affect reproductive hormones, follicular development, and ovarian aging [28]. The model based on systemic inflammatory indicators (CRP and complete blood count) showed the lowest predictive value (AUC = 0.58 ± 0.29) (Fig. 3C), highlighting that systemic inflammatory markers have limited predictive value for ovarian function, as diminished ovarian reserve appears to be more closely associated with localized pelvic microenvironmental shifts and chronic metabolic dysregulation rather than with overt systemic inflammation. To improve predictive sensitivity, we developed an integrated model combining microbial features and BMI, which achieved the highest performance (AUC = 0.88 ± 0.16) (Fig. 3D), offering a more comprehensive risk stratification approach for early detection of DOR.

These findings demonstrate that the integrated model, which combines pelvic microbial signatures with BMI, serves as an effective tool for both prediction and early diagnosis of diminished ovarian reserve, while also supporting individualized risk stratification through the inclusion of the metabolic indicator: BMI. Given the importance of translating early diagnostic insights into actionable preventive strategies [29], we next investigated the function of the identified microbial genera to explore their potential roles in ovarian health preservation.

### Functional potential of ascitic microbiota

**Fig. 4:**
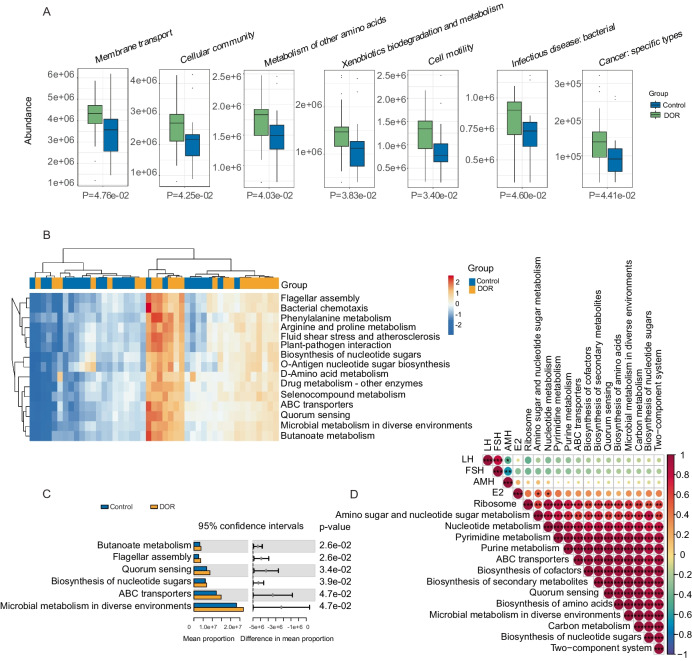
Functional potential of ascitic microbiota associated with DOR. **A** KEGG functional classification analysis of ascitic microbiota. **B** Differentially expressed KEGG pathways between the groups. **C** Top 6 differentially abundant KEGG pathways between the groups. **D** Correlation analysis between differential KEGG pathways and ovarian function markers (AMH, LH, FSH, E2) (control group: non-DOR group, AMH ≥ 1.1 ng/ml, DOR group representing AMH < 1.1 ng/ml) (correlation method: Spearman. Symbols represent significance levels: *P < 0.05, **P < 0.01, **P < 0.001, ***P < 0.0001)

With the correlation analysis results above, we found that pelvic microbiota dysbiosis is indeed a key factor influencing ovarian function. To investigate the functional potential of the ascitic microbiota, we first performed a Kyoto Encyclopedia of Genes and Genomes (KEGG) functional classification analysis to examine the microbial community’s overall functional profile. This analysis revealed a diverse array of pathways related to membrane transport, cellular community (prokaryotes), metabolism of other amino acids, xenobiotics biodegradation and metabolism, cell motility, infectious disease (bacterial), and cancer (specific types), indicating a potential impact of the microbiota on ovarian function (Fig. 4A). To further investigate the correlation between KEGG pathways and ovarian function, we analyzed the differentially enriched pathways the preserved ovarian function group and the DOR group (using AMH 1.1 ng/ml as the threshold, with control group representing AMH ≥ 1.1 ng/ml and DOR group representing AMH < 1.1 ng/ml). Following this, we found several KEGG pathways that differed significantly between the two groups. Specifically, microbial metabolism in diverse environments, adenosine triphosphate (ATP)-binding cassette (ABC) transporters, biosynthesis of nucleotide sugars, and quorum sensing were significantly enriched in the DOR group (Fig. 4B, C). These pathways may influence ovarian function and the aging process by regulating ovarian energy metabolism, DNA/RNA synthesis, cell proliferation, protein synthesis, and microenvironment modulation. We further analyzed the correlation between these differentially expressed pathways and ovarian function markers: AMH, LH, FSH, and E2. The results revealed that amino sugar and nucleotide sugar metabolism and nucleotide metabolism were positively correlated with E2, suggesting a potential role in estrogen biosynthesis, follicular development, and ovarian cellular function maintenance (Fig. 4D). These findings indicate that the identified microbial genera are not only useful for prediction and diagnosis but may also influence ovarian function through specific metabolic and endocrine-related pathways. Their potential involvement in hormone regulation and metabolic balance suggests that changes in the pelvic microbiota could contribute to diminished ovarian reserve. This highlights the value of developing preventive strategies that target microbiota-related dysregulation and supports the integration of microbial features into personalized clinical interventions.

## Discussion

This study highlights a novel diagnostic approach by investigating the association between the pelvic microbiome and ovarian reserve, an area that has received limited attention in reproductive medicine. While ovarian dysfunction has traditionally been associated with aging, DOR is now recognized as a multifactorial [30, 31] condition affecting diverse age groups. This is consistent with the concept of suboptimal health [32], in which functional deterioration precedes clinical symptoms, underscores the importance of early risk stratification and predictive diagnostics, which are key tenets of personalized reproductive care [33, 34]. Our results suggest that pelvic microbial alterations, particularly peritoneal shifts such as *Capnocytophaga* enrichment and loss of protective taxa, may act as early signals of ovarian reserve decline. This expands microbiome research from its traditional focus on the gut and vagina to include the broader pelvic microenvironment and supports a shift from symptom-based diagnosis toward individualized microbial risk assessment in reproductive care [16, 20, 35].

In our study, pelvic microbiome sequencing revealed reduced microbial diversity in younger women with DOR, suggesting that early dysbiosis may contribute to reproductive decline. Taxonomic analysis of ascitic fluid showed *Capnocytophaga* enrichment in the DOR group, while *Lautropia*, *Pelomonas*, and *Nesterenkonia* were more abundant in controls. Multivariate regression identified eight genera significantly associated with DOR risk, with *Capnocytophaga* showing the strongest effect. A microbiota-based model incorporating BMI showed improved accuracy, underscoring the combined value of metabolic and microbial markers in predicting ovarian dysfunction. To guide potential therapeutic strategies, KEGG pathway analysis revealed enrichment of quorum sensing, ABC transporters, and nucleotide biosynthesis in the DOR group. Pathways related to amino sugar and nucleotide metabolism were positively correlated with estrogen levels, suggesting microbial involvement in steroidogenesis and follicular development. These findings establish a mechanistic link between pelvic microbiota and ovarian aging, supporting early identification and personalized intervention. Our findings reveal early changes in the pelvic microbiota that may signal ovarian decline before symptoms appear. These microbial signatures may serve as earlier and more sensitive indicators than traditional clinical markers, enabling timely intervention. This represents a shift from delayed treatment to proactive prevention informed by microbiota profiling. Increasing evidence supports the use of probiotics, such as vaginal *Lactobacillus*, to restore microbial balance and maintain reproductive mucosal health, even in the absence of overt infection.

Specific probiotic strains differ significantly in their mechanisms of action and clinical relevance. For example, *Lactobacillus crispatus* has demonstrated strong mucosal adhesion, enhancement of epithelial barrier integrity, and suppression of pro-inflammatory cytokines such as interleukin-6 (IL-6) and tumor necrosis factor-alpha (TNF-α), making it a promising candidate for vaginal microbiota restoration [36]. In contrast, *Bifidobacterium longum* primarily exerts systemic metabolic and anti-inflammatory effects through short-chain fatty acid (SCFA) production and modulation of the gut–brain–ovary axis, thereby supporting hormonal regulation and ovarian endocrine function [37]. Therefore, when selecting effective probiotic candidates, it is essential to evaluate strain-specific properties such as acid and bile resistance, adhesion ability, carbohydrate fermentation capacity, and safety profiles, all of which determine their functional performance and suitability for personalized applications [38, 39]. Commonly studied oral strains include *B. breve*, *B. longum*, *L. rhamnosus*, and *L. reuteri*, all of which have shown benefits in modulating immunity, improving insulin sensitivity, and supporting reproductive hormonal balance [40].

The route of probiotic administration significantly influences therapeutic efficacy. Oral probiotics have systemic immunometabolic effects, supporting their role in managing DOR, polycystic ovary syndrome (PCOS), and infertility [40]. Prebiotics such as dietary fibers and nanoceria may enhance microbiota stability and boost therapeutic effects. These systemic strategies highlight the potential of microbiota-based therapies in reproductive health, though large multicenter studies are needed to confirm their clinical value [41, 42]. Intravaginal administration enables targeted modulation of the vaginal microbiota, facilitating rapid colonization by beneficial *Bacilli* strains and promoting local immune homeostasis. This route is particularly effective for managing vaginal dysbiosis and genitourinary syndrome of menopause (GSM), offering prompt symptom relief and epithelial restoration through direct microbial and mucosal interaction [43]. Combining systemic and local approaches may provide comprehensive benefits, particularly for patients with both metabolic risk and mucosal dysfunction.

Building on our findings and predictive model, we propose a clinically actionable framework for individualized intervention in patients with a BMI greater than 24.0 who are at increased risk of ovarian function decline. For those undergoing pelvic procedures, ascitic microbiota profiling may help identify early dysbiosis. If high-risk microbial signatures such as *Capnocytophaga* enrichment or depletion of protective taxa are identified, oral probiotics containing *Lactobacillus* and *Bifidobacterium* may help regulate metabolism and support reproductive health [40, 41]. Notably, personalized probiotic strategies that consider the unique microbial and metabolic profiles of each patient consistently outperform one-size-fits-all interventions, supporting a more predictive and personalized model of reproductive care [18, 38, 44]. Therapeutic strategies should be tailored to individual conditions. In human papillomavirus (HPV) related cases [45], restoring vaginal microbiota may support local immunity and reduce disease risk. For women with Flammer syndrome [46], personalized care addressing vascular and sensory factors is important. In genitourinary syndrome of menopause, when probiotics are not effective, non-hormonal options like carbon dioxide (CO_2_) laser or radiofrequency may relieve symptoms [47]. Tailoring treatment to a patient’s microbiota, hormones, and metabolism enables more precise and effective ovarian health management. Compared with generalized approaches, such individualized strategies offer greater clinical relevance and treatment efficiency.

This study introduces a predictive medical approach by identifying early microbial and metabolic indicators of ovarian reserve decline, particularly in high BMI individuals. For those with near-term fertility plans, ascitic microbiota profiling can be incorporated into routine diagnostic procedures such as laparoscopy or hysteroscopy. If microbial dysbiosis is detected, especially in individuals with enriched *Capnocytophaga* or depleted protective taxa, timely oral probiotic intervention may help reserve ovarian function. This personalized strategy supports early clinical action in metabolically vulnerable patients and bridges microbial diagnostics with fertility-preserving care. By identifying risk before clinical decline and enabling early personalized intervention, this study advances the shift from reactive management to predictive and preventive reproductive care in line with 3P medicine.

Nevertheless, our study is limited by its relatively small sample size and potential confounding factors, including pelvic history such as prior surgery or inflammatory disease. Its cross-sectional design also precludes causal inference. Moreover, although ascitic fluid provides valuable insights into the pelvic microenvironment, its clinical utility as a routine diagnostic marker is constrained by the invasive nature of sample collection. This underscores the urgent need to identify and validate non-invasive correlates, such as vaginal or urinary microbiota signatures. Future research should involve larger, ethnically diverse cohorts and adopt longitudinal designs to track microbial changes and their association with ovarian reserve over time. In addition, mechanistic studies using in vitro and in vivo models are needed to clarify causal relationships between specific microbes and ovarian function.

Progress in this field requires close collaboration among microbiologists, reproductive endocrinologists, immunologists, and bioinformaticians. Greater attention should also be given to the gut–liver–vagina–pelvis axis, which may underlie systemic microbial dysregulation linked to reproductive aging. Embracing this broader view may promote a shift toward earlier risk prediction, individualized prevention, and integrative management strategies for safeguarding ovarian function [19].

## Conclusions

This study underscores a significant association between the pelvic microbiome and ovarian reserve, particularly in women with DOR. Specific microbial taxa, such as *Capnocytophaga*, along with shifts in commensal populations, may contribute to ovarian dysfunction through chronic inflammation, metabolic stress, and hormonal dysregulation. These findings offer early mechanistic insights into subclinical changes that may lead to ovarian failure. By integrating pelvic microbiota profiles with BMI, we developed a predictive model to assess ovarian reserve risk. In individuals with BMI ≥ 24.0 and concurrent dysbiosis, targeted probiotic intervention may offer a personalized prevention strategy, illustrating the potential of predictive modeling to guide individualized care in line with 3P medicine. Our model offers a clinically applicable tool for early detection of ovarian risk, supporting proactive and personalized strategies before functional decline becomes evident. Strategies such as weight control, lifestyle modification, and probiotic use may help prevent premature ovarian insufficiency and support reproductive health. While our model provides a foundation for risk prediction and probiotic intervention, further research is required to validate its clinical utility and advance microbiota-targeted therapies. Developing non-invasive microbial biomarkers and personalized treatments for dysbiosis will be essential for improving early diagnosis and individualized prevention.

## Data Availability

Yes, I have research data to declare. The datasets generated and/or analyzed during the current study are available from the corresponding author on reasonable request.
